# Urine NMR-based TB metabolic fingerprinting for the diagnosis of TB in children

**DOI:** 10.1038/s41598-021-91545-0

**Published:** 2021-06-07

**Authors:** Patricia Comella-del-Barrio, José Luis Izquierdo-Garcia, Jacqueline Gautier, Mariette Jean Coute Doresca, Ramón Campos-Olivas, Clara M. Santiveri, Beatriz Muriel-Moreno, Cristina Prat-Aymerich, Rosa Abellana, Tomas M. Pérez-Porcuna, Luis E. Cuevas, Jesús Ruiz-Cabello, José Domínguez

**Affiliations:** 1 Institut d’Investigació Germans Trias i Pujol, Departament de Genètica i Microbiologia, Universitat Autònoma de Barcelona, Badalona, Barcelona, Spain; 2 CIBER de Enfermedades Respiratorias (CIBERES), Instituto de Salud Carlos III, Madrid, Spain; 3 Departamento de Química en Ciencias Farmacéuticas, Facultad de Farmacia, Universidad Complutense de Madrid, Madrid, Spain; 4 Cooperative Research in Biomaterials (CIC biomaGUNE), Basque Research and Technology Alliance (BRTA), Donostia, Spain; 5 Department of Pediatrics, Division of Tuberculosis, Hôpital Saint-Damien, Nos Petits-Frères Et Sœurs, Tabarre, Haiti; 6 Spectroscopy and Nuclear Magnetic Resonance Unit, CNIO Centro Nacional de Investigaciones Oncológicas, Madrid, Spain; 7 Julius Centre for Health Sciences and Primary Care, University Medical Center Utrecht, Utrecht University, Utrecht, The Netherlands; 8 Department of Basic Clinical Practice, Faculty of Medicine, University of Barcelona, Barcelona, Spain; 9 Servei de Pediatria, Atenció Primària, Unitat de Investigació Fundació Mútua Terrassa, Hospital Universitari Mútua Terrassa, Terrassa, Spain; 10 Department of Clinical Sciences, Liverpool School of Tropical Medicine, Liverpool, UK; 11 IKERBASQUE, Basque Foundation for Science, Bilbao, Spain

**Keywords:** Metabolomics, Tuberculosis, Diagnostic markers, NMR spectroscopy

## Abstract

Tuberculosis (TB) is a major cause of morbidity and mortality in children, and early diagnosis and treatment are crucial to reduce long-term morbidity and mortality. In this study, we explore whether urine nuclear magnetic resonance (NMR)-based metabolomics could be used to identify differences in the metabolic response of children with different diagnostic certainty of TB. We included 62 children with signs and symptoms of TB and 55 apparently healthy children. Six of the children with presumptive TB had bacteriologically confirmed TB, 52 children with unconfirmed TB, and 4 children with unlikely TB. Urine metabolic fingerprints were identified using high- and low-field proton NMR platforms and assessed with pattern recognition techniques such as principal components analysis and partial least squares discriminant analysis. We observed differences in the metabolic fingerprint of children with bacteriologically confirmed and unconfirmed TB compared to children with unlikely TB (p = 0.041 and p = 0.013, respectively). Moreover, children with unconfirmed TB with X-rays compatible with TB showed differences in the metabolic fingerprint compared to children with non-pathological X-rays (p = 0.009). Differences in the metabolic fingerprint in children with different diagnostic certainty of TB could contribute to a more accurate characterisation of TB in the paediatric population. The use of metabolomics could be useful to improve the prediction of TB progression and diagnosis in children.

## Introduction

One-quarter of the world’s population is infected with *Mycobacterium tuberculosis,* and 10 million people fell ill with tuberculosis (TB) in 2019^1^. TB is also a major cause of morbidity and mortality in children, with an estimated one million dying from TB each year^1^. Under-detection of childhood TB is common in low- and middle-income countries^2^ as its clinical presentation overlaps with other respiratory infections, children have low sputum bacillary loads and are often unable to produce sputum, making its diagnosis difficult^3,4^.

Metabolomics, or the systematic study of a unique chemical fingerprint present in a cellular system or biofluid, increasingly allows discrimination between samples with different physiological or pathological states^5^. These fingerprints can be measured in biological samples, such as urine, serum or plasma, using non-invasive methods such as nuclear magnetic resonance (NMR) spectroscopy^6^, and have been used to monitor metabolic changes over time induced by pathogens^7^. Furthermore, the application of metabolomics to low-field (LF) NMR spectrometry has facilitated the development of smaller platforms suitable for primary and secondary medical centres laboratories^8,9^. In recent years, metabolomics has facilitated gaining insights into TB pathogenesis^10^, disease progression, and evaluation of treatment responses^11^.

A few studies have focused on the discovery of urine-based biomarkers for TB diagnosis. Urine is a non-invasive sample that requires minimal preparation^12^ and would be an easily obtained clinical sample for diagnosis, especially for individuals unable to produce sputum, such as children. In this study, we aimed to describe a urine proton (1H) NMR-based metabolic fingerprint for the diagnosis of TB in children.

## Results

One hundred and seventeen children were included, of which 62 had presumptive TB and 55 were apparently healthy (controls). Sixty-eight (58.1%) were male, and their mean (SD) age was 7 (3.6) years (Table 1). There were no sex or age differences between children with presumptive TB and controls. Eighty-one (69.2%) participants had received the Bacillus Calmette et Guérin (BCG) vaccine and had a BCG scar (Table 1). Among the 62 children with presumptive TB, 6 had bacteriologically confirmed TB, 52 unconfirmed TB (bacteriologically negative) and four were considered to be unlikely to have TB (unlikely TB), as described in Table 2. Eighteen (29%) of the 62 children with presumptive TB had X-rays compatible with intrathoracic TB, nine (14.5%) had X-rays and clinical findings of extra-thoracic TB, and four (6.5%) had both intra- and extra-thoracic TB. Thirty-one (50%) children’s X-rays were considered inconclusive. Fifty-seven (91.9%) children had positive tuberculin skin test (TST, 54, 88.5%) and/or QuantiFERON-TB Gold In-Tube test (QFT-GIT, 39, 70.9%), with 63.2% (36/57) agreement between the tests. Fifty-three (85.5%) had documented exposure to an index case of TB. Seven (11.3%) of the 62 children had five clinical criteria of TB, while 21 (33.9%) had four, 28 (45.2%) had three, and six (9.7%) had two clinical criteria.

**Table 1:** Demographic and clinical characteristics of the study participants.

Variable	All (n = 117)	Presumptive TB (n = 62)	Controls (n = 55)	p-value
**Gender**				0.717
Girls	49 (41.9%)	25 (40.3%)	24 (43.6%)	
Boys	68 (58.1%)	37 (59.7%)	31 (56.4%)	
**Age in years**				
Mean (SD)	6.89 (3.56)	7.28 (4.05)	6.45 (2.90)	0.203
**Range**				0.640
≤ 5	40 (34.2%)	20 (32.3%)	20 (36.4%)	
> 5	77 (65.8%)	42 (67.7%)	35 (63.6%)	
**BCG scar**				0.259
Yes	81 (69.2%)	43 (69.4%)	38 (69.1%)	
No	30 (25.6%)	14 (22.6%)	16 (29.1%)	
Unknown	6 (5.1%)	5 (8.1%)	1 (1.8%)	

Categorical variables expressed as number of subjects (n) and percentage (%), and quantitative variables expressed as median and standard deviation (SD).

*TB* tuberculosis, *BCG* Bacillus Calmette-Guérin.

**Table 2:** Demographic information and clinical criteria of children with presumptive TB.

Variable	All (n = 62)		Confirmed TB (n = 6)	Unconfirmed TB (n = 52)	Unlikely TB (n = 4)		p-value
**Gender**							0.742
Girls	25 (40.3%)		2 (33.3%)	22 (42.3%)	1 (25.0%)		
Boys	37 (59.7%)		4 (66.7%)	30 (57.7%)	3 (75.0%)		
**Age in years**							
Mean (SD)	7.3 (4.1)		6.7 (3.9)	7.0 (4.0)	11.6 (3.3)		0.090
**Range**							0.250
≤ 5	20 (32.3%)		3 (50.0%)	17 (32.7%)	0 (0.0%)		
> 5	42 (67.7%)		3 (50.0%)	35 (67.3%)	4 (100.0%)		
**BCG scar**							0.540
Yes	43 (69.4%)		4 (66.7%)	37 (71.2%)	2 (50.0%)		
No	14 (22.6%)		2 (33.3%)	10 (19.2%)	2 (50.0%)		
Unknown	5 (8.1%)		0 (0.0%)	5 (9.6%)	0 (0.0%)		
**TB type**							** < 0.001**
Intrathoracic	18 (29.0%)		2 (33.3%)	16 (30.8%)	0 (0.0%)		
Extrathoracic	9 (14.5%)		1 (16.7%)	8 (15.4%)	0 (0.0%)		
Both	4 (6.5%)		3 (50.0%)^a, b^	1 (1.9%)^a^	0 (0.0%)^b^		
Not defined	31 (50.0%)		0 (0.0%)^a, b^	27 (51.9%)^a^	4 (100.0%)^b^		
**Immunologic evidence of *M. tuberculosis* infection**							0.619
Yes	57 (91.9%)		5 (83.3%)	48 (92.3%)	4 (100.0%)		
No	5 (8.1%)		1 (16.7%)	4 (7.7%)	0 (0.0%)		
**TB exposure**							**0.001**
Yes	53 (85.5%)		2 (33.3%)^a, b^	47 (90.4%)^a^	4 (100.0%)^b^		
No	9 (14.5%)		4 (66.7%)^a, b^	5 (9.6%)^a^	0 (0.0%)^b^		
**Symptoms/signs suggestive of TB**							**0.006**
≤ 2	32 (51.6%)		0 (0.0%)^a, b^	28 (53.8%)^a^	4 (100.0%)^b^		
≥ 3	30 (48.4%)		6 (100.0%)^a, b^	24 (56.2%)^a^	0 (0.0%)^b^		
**Lymphadenopathy**							0.092
Yes	22 (35.5%)		4 (66.7%)	18 (34.6%)	0 (0.0%)		
No	40 (64.5%)		2 (33.3%)	34 (65.4%)	4 (100.0%)		
**Chest radiograph**							**0.006**
Abnormal	30 (48.4%)		6 (100.0%)^a, b^	24 (46.2%)^a^	0 (0.0%)^b^		
Normal	32 (51.6%)		0 (0.0%)^a, b^	28 (53.8%)^a^	4 (100.0%)^b^		
**Response to TB treatment**							** < 0.001**
Treatment completed	52 (83.9%)		5 (83.3%)^a^	47 (90.4%)^b^	0 (0.0%)^a, b^		
Lost to follow-up	8 (12.9%)		0 (0.0%)^a^	4 (7.7%)^b^	4 (100.0%)^a, b^		
Died	2 (3.2%)		1 (16.7%)	1 (1.9%)	0 (0.0%)		
**Anti-TB treatment or preventive treatment**							0.302
Under treatment	24 (38.7%)		4 (66.7%)	19 (36.5%)	1 (25.0%)		
Untreated	38 (61.3%)		2 (33.3%)	33 (63.5%)	3 (75.0%)		

^a,b^Significant differences in variables when comparing proportions between study groups. If a pair of values is significantly different, the values have the same superscript letters assigned to them. Bold values, significative statistical values with a p-value under 0.05. Categorical variables expressed as number of subjects (n) and percentage (%), and quantitative variables expressed as median and standard deviation (SD).

*TB* tuberculosis, *BCG* Bacillus Calmette-Guérin.

Thirty-one (43.6%) of the 55 controls were male, and their mean (SD) age was 6.5 (2.9) years, as shown in Table 1.

### Performance of the TB metabolic fingerprinting

**Figure 1:**
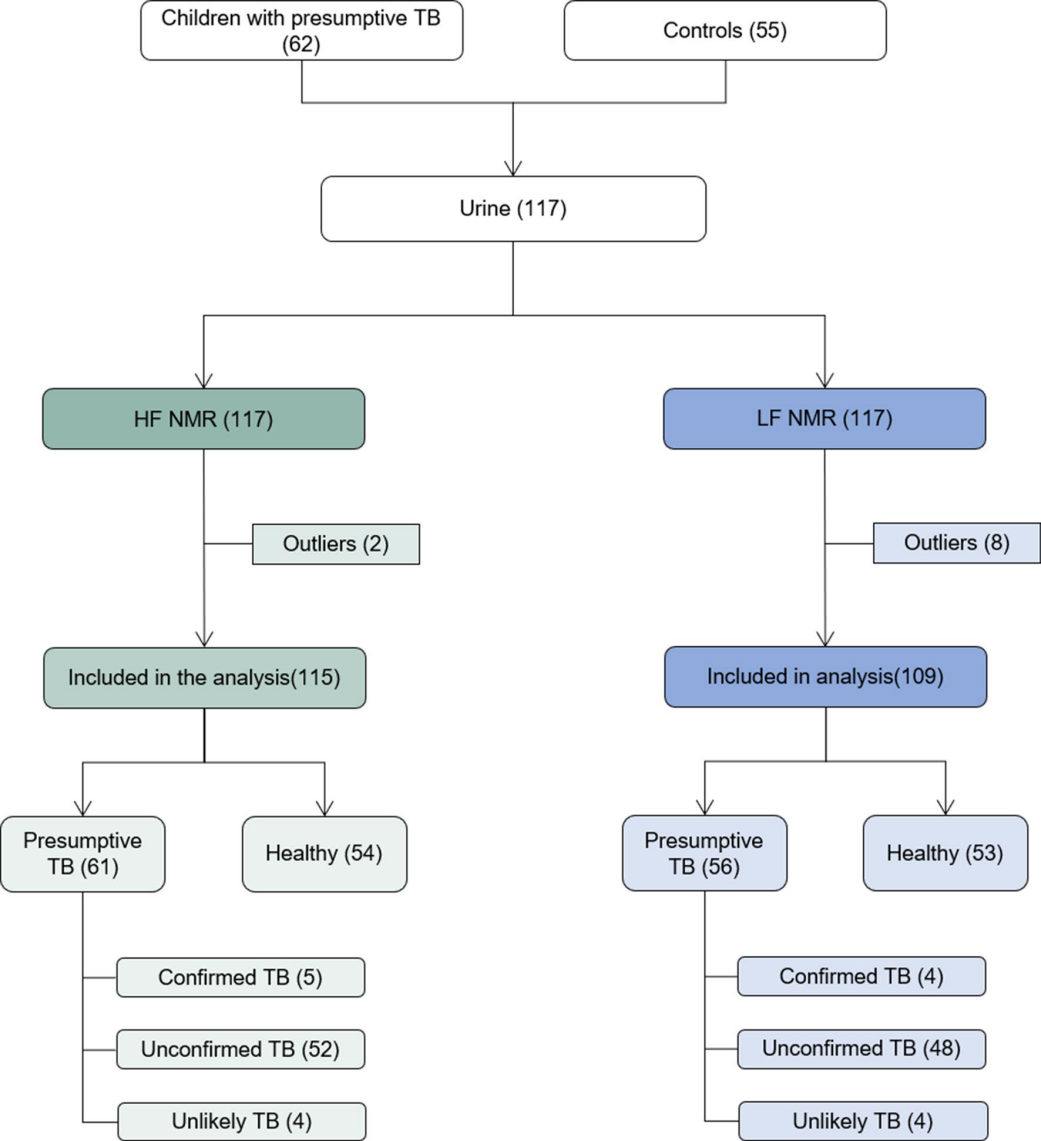
Description of the children who participated in the study according to the nuclear magnetic resonance equipment used to acquire the urine samples spectra and the classification of the patients in the study group. *NMR* nuclear magnetic resonance, *HF* high-field, *LF* low-field, *TB* tuberculosis.

The metabolic fingerprint of the urine samples (n = 117) were measured using both high-field (HF) and LF 1H NMR spectroscopy, as detailed in Fig. 1. Representative spectra obtained with the HF and LF 1H NMRs are shown in Supplementary Fig. 1.

**Figure 2:**
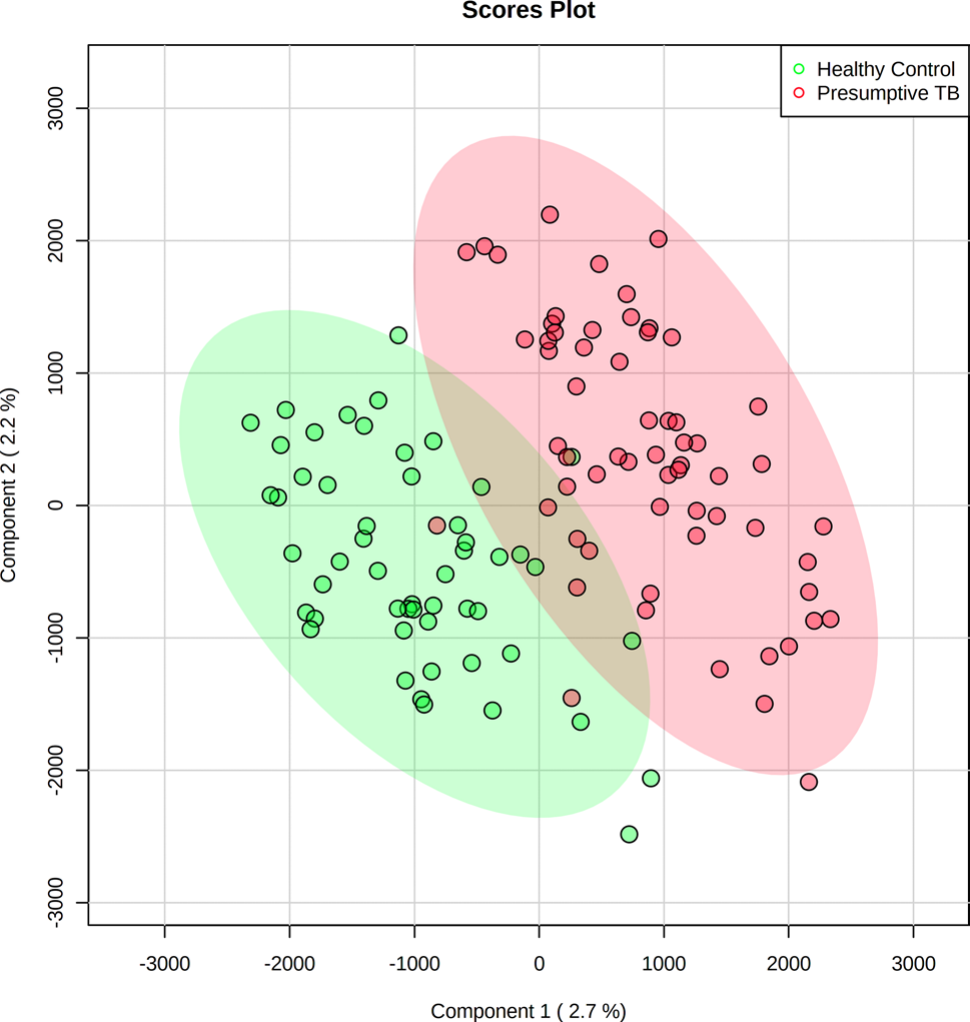
Partial least squares discriminant analysis (PLS-DA) score plot of urine spectra measured using high-field proton (1H) nuclear magnetic resonance spectroscopy of children with presumptive TB (n = 61) and healthy children (n = 54). Two-dimensional view showing the distribution of the groups according to the first two components of the PLS-DA model. TB, tuberculosis. Metaboanalyst 5.0. (https://www.metaboanalyst.ca).

**Figure 3:**
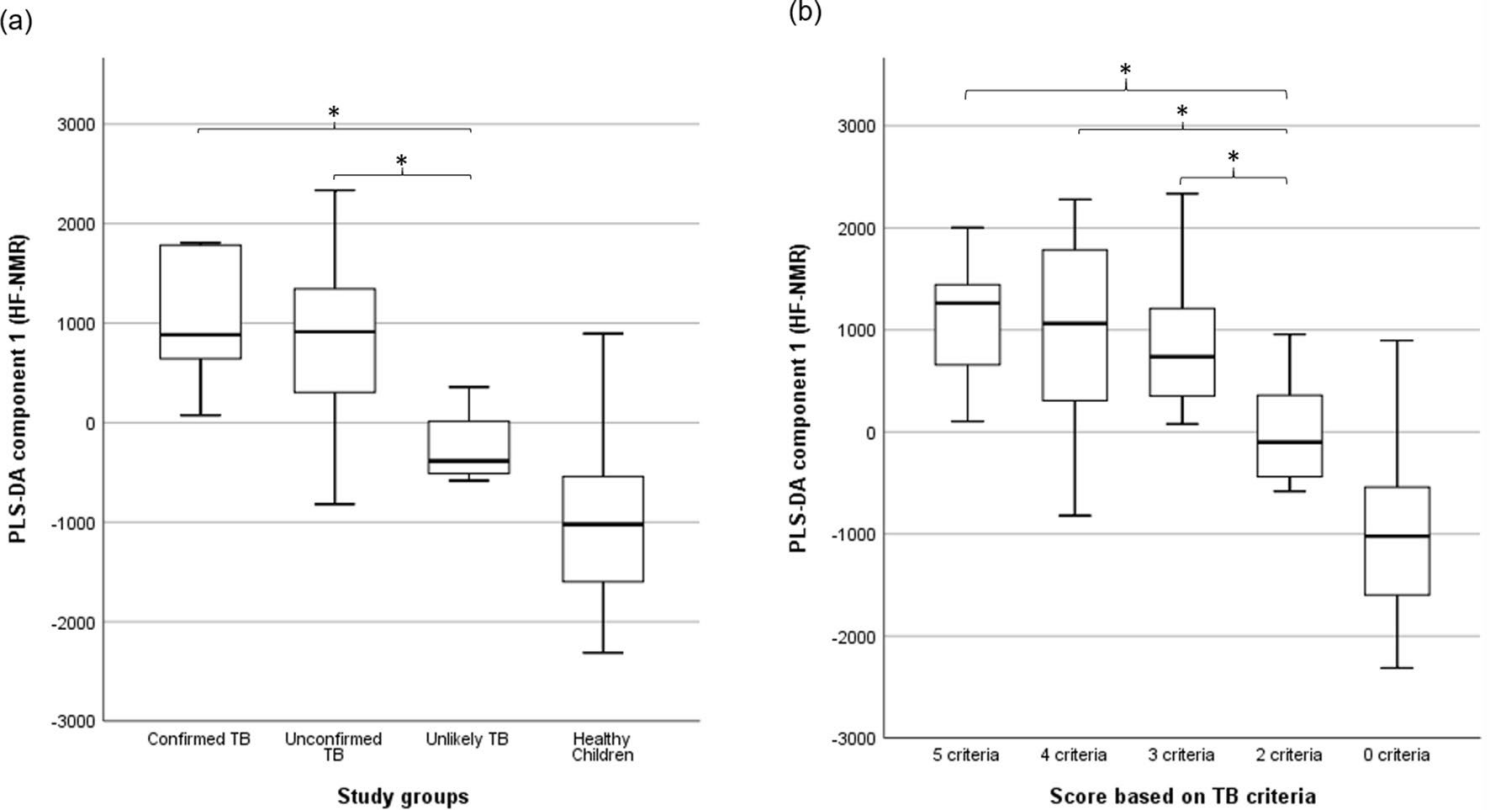
Association between partial least squares discriminant analysis (PLS-DA) scores and (**a**) study groups, and (**b**) the number of clinical criteria compatible with TB in 115 urine spectra acquired by high-field proton (1H) nuclear magnetic resonance. The central horizontal line within the boxes represents the median. The boxes comprise the first and third quartiles, the tiles indicate the maximum and minimum values, and the asterisk indicates statistically significant differences (p-value < 0.05) between groups. TB: tuberculosis; PLS-DA 1, the first component of the PLS-DA model**.** IBM SPSS Statistics 26 (https://www.ibm.com).

An unsupervised principal component analysis (PCA)^13^ was applied to the HF 1H NMR urine spectra of the six bacteriologically confirmed, 52 unconfirmed and four unlikely TB, and the 55 controls not showing clustering patterns between samples. Two children’s samples (one bacteriologically confirmed TB and one control) were considered outliers in the PCA score plot and were excluded^14^ (Supplementary Fig. 2). A supervised partial least squares discriminant analysis (PLS-DA) was applied to identify a discriminatory metabolic pattern between presumptive TB and control groups to the remaining 115 urine samples. We observed groupings along the scores of the first component of the PLS-DA (PLS-DA component 1) (Fig. 2). The robustness parameters of the HF PLS-DA model were tested by Leave-One-Out Cross-Validation (LOOCV) using the PLS-DA component 1 showing a performance accuracy to discriminate between presumptive TB and controls of 0.68, with R2 and Q2 values of 0.61 and 0.13, respectively; and an Area Under the Curve of Receiver Operating Characteristic (AUC-ROC) of 0.65. The Variable Importance in Projection (VIP) scores for the PLS-DA component 1 identified the main spectral regions of the metabolic fingerprint to differentiate between children with presumptive TB and controls (Supplementary Table 1). There was a trend in the PLS-DA component 1 scores with the certainty of TB diagnosis (Fig. 2). Thus, children with bacteriologically confirmed (n = 5) and unconfirmed TB (n = 52) had higher median PLS-DA component 1 scores (883.3 ± 751.1 and 913.3 ± 716.6) than children with unlikely TB (n = 4; − 385.2 ± 417.3) (p = 0.026 and p = 0.005, respectively; Fig. 3a). The PLS-DA component 1 scores also varied with the number of clinical criteria for TB. Children with five (n = 7), four (n = 21), and three (n = 27) clinical criteria had significantly higher median PLS-DA component 1 scores (1262.4 ± 649.8, 1062.7 ± 839.3, and 736.3 ± 644.6, respectively) than children with two (n = 6) criteria (− 100.5 ± 582.7) (p = 0.014, p = 0.007, and p = 0.021, respectively; Fig. 3b). Children with unconfirmed TB with X-rays compatible with TB (n = 24) had higher PLS-DA component 1 scores than unconfirmed cases with normal X-rays (n = 28) (1080.8 ± 736.3 and 796.2 ± 634.4) (p = 0.043).

**Figure 4:**
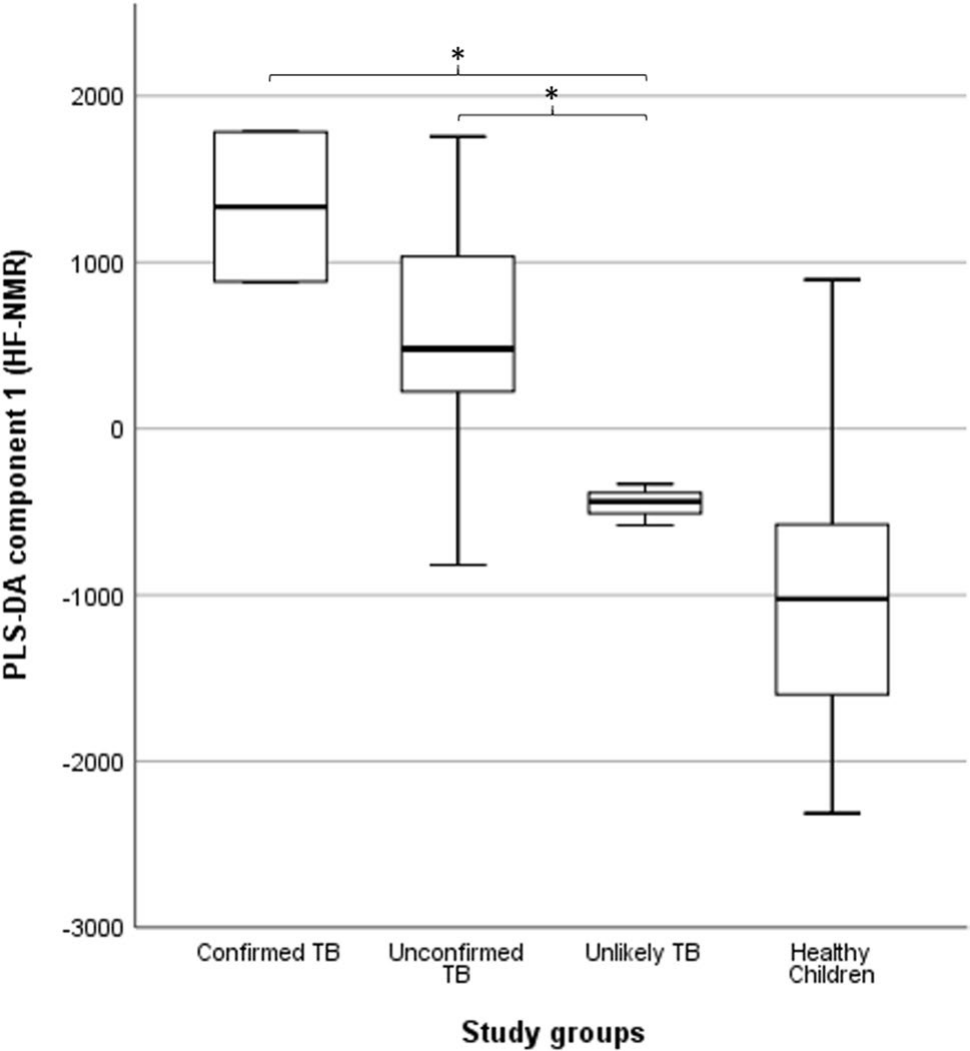
Association between partial least squares discriminant analysis (PLS-DA) scores and study groups in 92 urine spectra of children without TB-treatment acquired by high-field proton (1H) nuclear magnetic resonance. The central horizontal line within the boxes represents the median. The boxes comprise the first and third quartiles, the tiles indicate the maximum and minimum values, and the asterisk indicates statistically significant differences (p-value < 0.05) between groups. *TB* tuberculosis; PLS-DA 1, the first component of the PLS-DA model. IBM SPSS Statistics 26 (https://www.ibm.com).

If children who had already started treatment (for less than 15 days) are excluded from the analysis (4 confirmed TB, 19 unconfirmed TB and 1 unlikely TB), children with bacteriologically confirmed TB or unconfirmed TB still had significantly higher median PLS-DA component 1 scores (1333.4 ± 636.5 and 480.3 ± 508.5, respectively) than children with unlikely TB (− 437.9 ± 125.1) (p = 0.005 and p = 0.010, respectively; Fig. 4).

**Figure 5:**
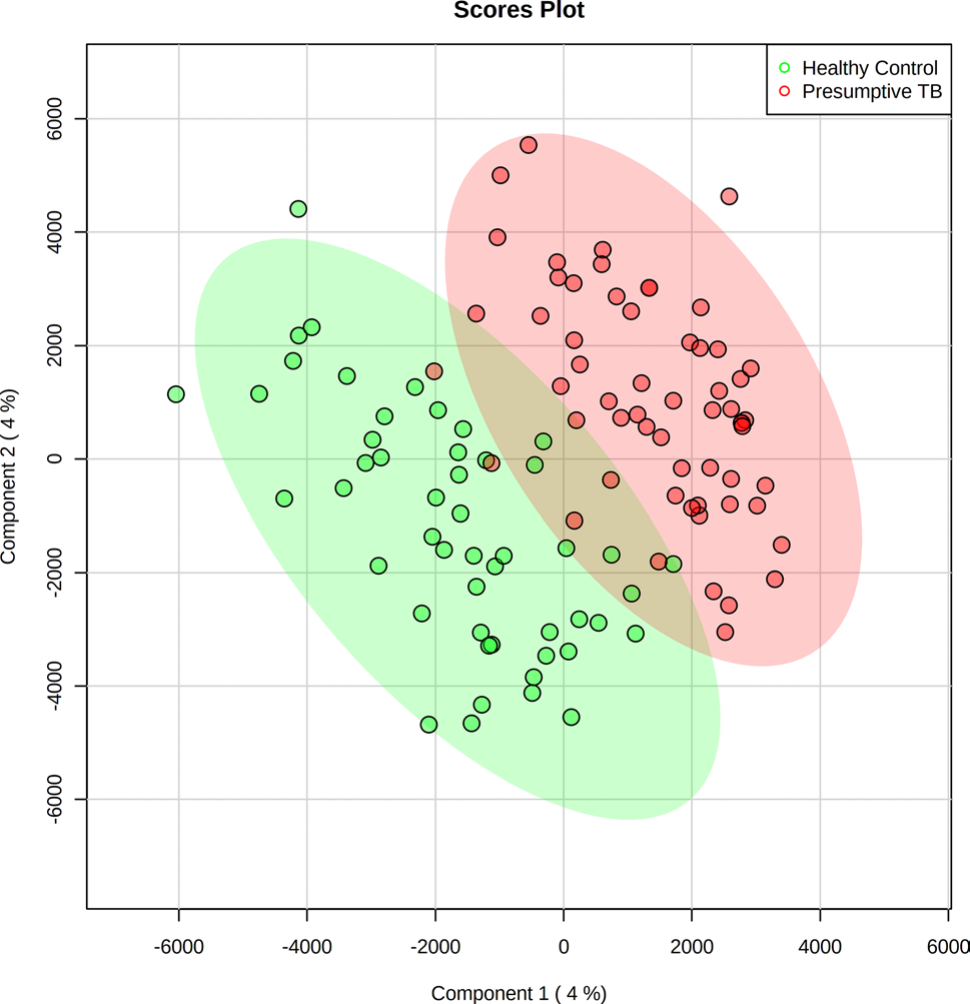
Partial least squares discriminant analysis (PLS-DA) score plot of urine spectra measured using low-field proton (1H) nuclear magnetic resonance spectroscopy of children with presumptive TB (n = 56) and healthy children (n = 53). Two-dimensional view showing the distribution of the groups according to the first two components of the PLS-DA model. TB, tuberculosis. Metaboanalyst 5.0. (https://www.metaboanalyst.ca).

**Figure 6:**
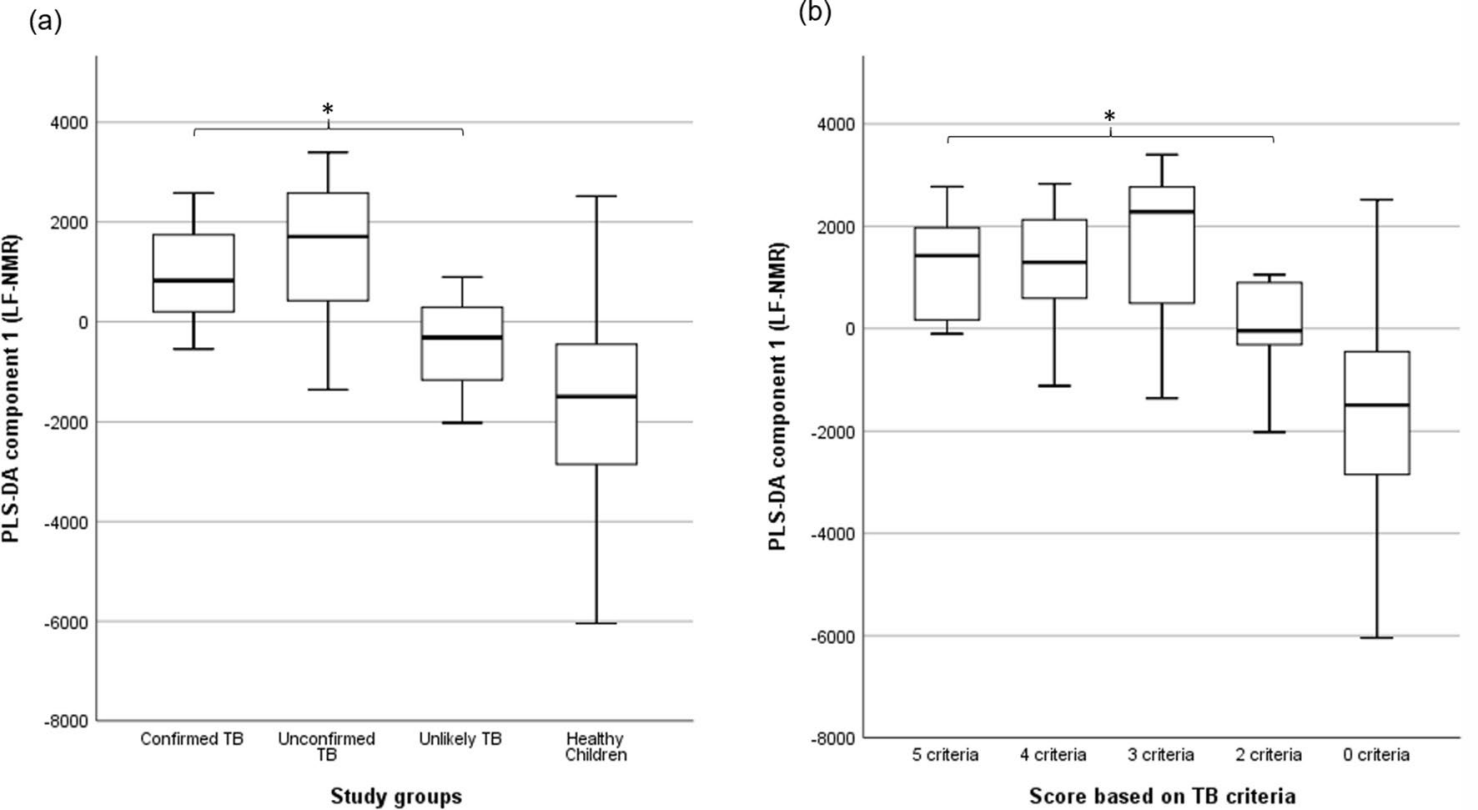
Association between partial least squares discriminant analysis (PLS-DA) scores and (**a**) study groups, and (**b**) the number of clinical criteria compatible with TB in 109 urine spectra acquired by low-field proton (1H) nuclear magnetic resonance. The central horizontal line within the boxes represents the median. The boxes comprise the first and third quartiles, the tiles indicate the maximum and minimum values, and the asterisk indicates statistically significant differences (p-value < 0.05) between groups. TB: tuberculosis; PLS-DA 2, the second components of the PLS-DA model**.** IBM SPSS Statistics 26 (https://www.ibm.com).

The PCA applied to the 117 LF 1H NMR acquired urine fingerprints detected eight outliers (two bacteriologically confirmed TB, 4 unconfirmed TB and two controls), which were excluded from the PLS-DA (Supplementary Fig. 3). A PLS-DA was applied to the remaining 109 urine fingerprints to identify a discriminatory metabolomic pattern between presumptive TB and control groups. We observed groupings between presumptive TB and control groups along the scores of the first component of the PLS-DA (PLS-DA component 1) (Fig. 5). PLS-DA component 1 scores were higher in children with presumptive TB than controls. The robustness parameters of the LF PLS-DA model were tested by LOOCV using the PLS-DA component 1 (performance accuracy to discriminate between presumptive TB and controls = 0.70, R2 = 0.76, Q2 = 0.08, and AUC-ROC = 0.65). Supplementary Table 1 shows the VIP for PLS-DA component 1 responsible for differentiating between children with presumptive TB and controls. Children with bacteriologically confirmed TB (n = 5) had higher median PLS-DA component 1 scores (825.2 ± 1236.52) than children with unlikely TB (n = 3; − 316.5 ± 1464.3) (p = 0.040 Fig. 6a). The PLS-DA component 1 scores also varied with the number of clinical criteria for TB. Children with five (n = 6) clinical criteria had significantly higher median PLS-DA component 1 scores (1426.1 ± 1088.1) than children with two (n = 5) criteria (− 46.3 ± 1229.8) (p = 0.009; Fig. 6b). The median PLS-DA component 1 scores among children with unconfirmed TB with compatible TB X-rays (n = 23) and normal X-rays (n = 28) were similar (1518.7 ± 1136.3 and 1920.1 ± 1419.3) (p = 0.643).

## Discussion

Early diagnosis and treatment are crucial to stop the epidemic of childhood TB^2^. The search for biomarkers in non-invasive biological samples as alternatives to sputum is needed to improve the diagnostic sensitivity of TB in this population^1,15^. We report here a urine NMR-based metabolic fingerprint associated with bacteriologically and clinically diagnosed TB in children^16^.

Recently, the detection of TB from Xpert MTB/RIF (Cepheid, Sunnyvale, CA, USA) in urine has been evaluated in children^17^; however, the results have not achieved the accuracy desired to improve TB diagnosis in this population. Other emerging alternative diagnostic include Fujifilm SILVAMP TB (FujiLAM, Fujifilm, Tokyo, Japan), a new assay generation for detecting lipoarabinomannan (LAM) in urine^18,19^. In studies evaluating FujiLAM in children, the sensitivity and specificity reported were 50% and 92%, respectively, in a South African cohort, and 64.9% and 83.8%, respectively, in a multicentre evaluation in Africa ^20,21^. Despite its low sensitivity, its high specificity could help confirm the disease in children with a high probability of TB (e.g. children living in high-TB burden areas and those with HIV or malnutrition).

Biofluid metabolomics provides a snapshot of all the mechanisms that act during the disease, thus facilitating understanding the interaction between host and pathogen during infection and TB disease progression^22,23^. Previously, metabolomic profiles have been described in serum^24–29^ and plasma^24,25,30,31^ by NMR spectroscopy and mass spectrometry for the prediction and detection of TB. In children, two studies have reported different metabolomic profiles for TB detection in plasma^32^ and serum^33^ analysed by 1H NMR spectroscopy. However, neither of these studies reported whether the metabolic profile could discriminate between children with different diagnostic certainty of TB^32^. We have previously identified an NMR‑based metabolomic profile in urine to diagnose TB in adults^34^ and here we extend their potential application to the diagnosis of TB in a paediatric population and we have demonstrated differences in the urinary metabolic fingerprint of children with different certainty in the TB diagnosis.

TB in children is characterized by a continuum of conditions correlated with bacterial load, host immune responses, clinical manifestation, and the detection of *M. tuberculosis*^3^. Inflammatory host biomarkers in plasma have potential to discriminate latent TB infection from overt TB in children, and to identify the onset of TB disease ^35–37^. During latent infection, the host is able to contain the infection, the bacteria has restricted metabolic activity and there are no clinical manifestations. However, with progression to active TB, the infection overcomes the host immune system, the bacilli replicate, and the increased metabolomic activity of the mycobacteria modifies the tissues physiopathology, with changes in the host metabolome. The metabolic fingerprinting analysed by HF 1H NMR spectroscopy showed metabolic differences between children with presumptive TB with two or fewer clinical criteria and three or more clinical criteria. Moreover, children with high diagnostic certainty of TB showed metabolic fingerprints similar to that of children with bacteriologically confirmed TB. This metabolic response could be attributed to the physiological stimuli that occurs during disease progression^23,38^.

The paucibacillary nature of TB in children, combined with the limitation of current microbiological methods, results on a high dependence on chest X-rays for diagnosis^39^. In this study, children with unconfirmed TB and abnormal X-rays had differences in their metabolic fingerprint compared with those with normal X-rays. The differences in the metabolic fingerprint are consistent with studies interpreting the occurrence of radiological features from the pathway of incipient TB infection to subclinical and symptomatic TB^40,41^.

One limitation of this study is the low confirmation rate of TB (8% and 7% in HF and LF NMR metabolic fingerprint approach, respectively). This low rate, together with the inherent resonance overlap phenomenon in LF spectrometers (60 Hz)^8,9^ might have hindered the pattern recognition process in the LF metabolic fingerprinting approach, losing its discriminatory power between the study sub-groups with presumptive TB. The compact and portable size and the successful performance of this approach, demonstrated in previous studies^8,9,34^, makes LF benchtop NMR-based metabolic fingerprinting a promising diagnostic tool. However, further analysis with a larger group of children with confirmed TB is needed to evaluate the full potential of this approach in children as the small final number of bacteriologically confirmed TB cases (9.7%) in our study, prevented the development of a TB-specific discriminatory model.

In summary, this study identified an association between the urine NMR-based metabolic fingerprint and the clinical case definitions used for the classification of TB in children, and observed differences in the metabolic response of children with different diagnostic certainty of TB. This finding could contribute to the identification and classification of childhood TB, which would improve the characterization of the clinical spectrum of the disease and the search for new diagnostic and prognostic biomarkers of TB in children.

## Methods

This was a prospective case series of children aged 0 to 14 years old with presumptive TB attending the St. Damien Paediatric Hospital, Port-au-Prince, Haiti, in 2015 and 2016, and healthy children attending a local primary school in the same neighbourhood.

Clinical and demographic information obtained at the time of enrolment included age, sex, weight, medical history and clinical presentation (history and exposure of TB, presence of cough, fever for ≥ 2 weeks, unexplained weight loss, and asthenia/fatigue, TB treatment, HIV status, and comorbidities), vaccines received (including BCG), and current and previous medications. Children with known immunodeficiencies, those receiving immunosuppressive treatment, or those starting TB treatment more than 15 days ago were excluded. The Mantoux TST (Sanofi Pasteur, Canada) and the QFN-GIT (Qiagen, Germany) assays were performed and interpreted according to the manufacturer’s instructions.

All children with presumptive TB had a chest X-ray and induced or aspirated nasopharyngeal/nasogastric sputum collected on three consecutive days. Sputum was examined using fluorescent smear microscopy (auramine stain). Children with positive smear microscopy or abnormal X-rays were tested with Xpert MTB/RIF. Children with lymph node adenopathy underwent biopsies, and specimens underwent histological examination from a pathologist.

Children with presumptive TB were classified, following the updated clinical case definitions for classification of intrathoracic TB in children into confirmed, unconfirmed, and unlikely TB^16^. Children were classified as: confirmed TB, if bacteriological confirmation was attained by Xpert MTB/RIF; unconfirmed TB, if there was not bacteriological confirmation, but evidence of *M. tuberculosis* infection (i.e., TST or QFT-GIT positive) and at least one clinical criteria of the clinical case definition (i.e., X-ray consistent with TB, symptoms and signs suggestive of TB, close TB exposure, or positive response on TB treatment), or two clinical criteria, if TST and QFT-GIT results were negative. Children were considered unlikely TB if the child had only evidence of *M. tuberculosis* infection or presented only one clinical criterion compatible with TB. School children were enrolled as controls if they had negative TST and QFT-GIT and no signs or symptoms of TB.

### Urine collection

Midstream urine samples were collected from all participants in sterile plastic containers following standardized procedures^12^. In children who attended the hospital, urine samples were collected within the first week of the TB diagnosis. Two millilitres of urine were aliquoted in cryovials with screw caps that were frozen at − 20 °C until the 1H NMR analysis. According to a protocol established in a previous study^34^, 400 µl of urine were mixed with either 200 µl of the standard deuterated buffer for HF 1H NMR measurements or 250 µl for LF 1H NMR measurements. The standard deuterated buffer was a 0.2 M phosphate buffer solution dissolved in 99.9% deuterated water to adjust the internal pH to 7.4, containing 0.09% sodium azide and 0.3 mM trimethylsilyl propanoic acid (TMSP). Six hundred µl of buffered urine was transferred into 5 mm diameter NMR tubes (CortecNet, Les Ulis, France) for 1H NMR spectra acquisition.

### Acquisition of NMR spectra

All 1H NMR urine spectra were measured following the procedures previously described^9,42^ using two different instruments operating at HF and LF, respectively: (1) a Bruker Avance 700 MHz spectrometer at a 1H frequency of 700 MHz (CNIO, Madrid, Spain) and (2) a Magritek Spinsolve 60 Ultra benchtop NMR spectrometer at a 1H frequency of 60 MHz (Magritek GmbH, Aachen, Germany). Briefly, HF 1H NMR spectra were measured using a pulse sequence based on the first increment of the nuclear Overhauser effect spectroscopy (NOESY) with pre-saturation to effect suppression of the water signal (δ =  ~ 4.80 ppm). The spectra were acquired using the following parameters: 32,000 data points over a spectral width of 8,333.33 Hz and 256 scans resulting in acquisition times of 13 min per sample. LF 1H NMR spectra were measured using a one-dimensional presaturation (1D PRESAT) sequence to allow for efficient saturation of the water signal (δ =  ~ 4.95 ppm). The spectra were acquired using the following parameters: 64 scans, an acquisition time of 6.4 s, and shimming of the sample after each new one to maintain a line width below 0.55 Hz. Data were zero-filled before Fourier transformation, and free induction decays (FIDs) were multiplied by exponential line broadening of 0.3 Hz.

### Processing spectral data

Spectral data were processed using the MestReNova program (v.14; Mestrelab Research, Santiago de Compostela, Spain). According to the established protocols described in previous studies^34,42^, metabolite signals of the spectra were shift-aligned using TMSP as a reference signal standard (δ = 0.00 ppm), and the chemical shift regions of the raw HF 1H NMR spectra from 6.50 to 4.22 ppm were excluded from the analysis to remove the random effects of variation in urea and water resonance suppression^34,42^. Then, the chemical shift region around 0.00 ppm containing the internal reference (TMSP) was excluded, and baseline correction was performed using the ‘Withakker Smoother’ algorithm^34,42^. Binning (also known as bucketing) was applied to 1H NMR spectra and data-reduced to equal length integral segments (bins) of 0.04 ppm to compensate variations in resonance positions. All bins were normalized by the total sum of the spectral regions (each bin was divided by the sum of all the 1H NMR signals) ^34,42^. Thus, the concentration of each metabolite was normalized by the urine total metabolite content to compare these concentrations (in arbitrary units) between samples^34,42^. Before multivariate statistical analysis, spectral data were Pareto scaled^43^, where the square root of the standard deviation is used as the scaling factor.

### Multivariate analysis of spectral data

Processed 1H NMR data were analysed in a multivariate manner using the Metabonomic package of R software (rel.3.3.1)^44,45^ and MetaboAnalyst v.5.0^46^. The analysis included all urine spectra acquired using the HF and LF 1H NMR equipment. Graphs were plotted using SPSS statistical software for windows (SPSS version 26; SPSS Inc, Chicago, IL, USA).

Unsupervised data were analysed by applying the PCA to reduce the dimensionality of NMR-processed data and to observe clustering patterns according to their elemental composition^13,14^. In addition, PCA score plots were used to highlight statistical outliers based on Mahalanobis distance. Mahalanobis distance was calculated from the data point to the centroid of all samples in PC1, PC2, and PC3 three-dimensional space. A single case was considered a statistical outlier if it was placed out of the tolerance ellipse of 97.5%^47^.

Supervised PLS-DA^13^ was applied to the metabolic fingerprint of children with presumptive TB and controls to detect a discriminatory metabolic pattern between groups. Thus, all spectral regions grouped in bins of 0.04 ppm were transformed into a new set of orthogonal components obtained by maximising the covariance between spectral data and the class membership (presumptive TB and controls).

The robustness of the HF and LF PLS-DA models using the PLS-DA component 1 was validated using the LOOCV procedure (performance accuracy, R2, Q2, AUC-ROC). The VIP scores for PLS-DA were calculated to identify the spectral regions of the metabolic fingerprint most important for differentiating between children with presumptive TB and controls. Statistical significance was determined using Student’s t-test.

Since PLS-DA scores were trained to maximise the covariance between spectral data and class membership (presumptive TB vs controls), we hypothesised that the same PLS-DA scores should be sensitive also to differences within the group of children with presumptive TB (sub-categorised into bacteriologically confirmed TB, unconfirmed TB, and unlikely TB). Thus, the resulting PLS-DA component 1 (first latent variable) scores were used to evaluate metabolic differences between children with presumptive TB classified according to the standardized case definitions for TB and with the number of clinical criteria of TB.

### Statistical analysis

Clinical and demographic characteristics were described using descriptive statistics. Categorical variables were described using frequencies and percentages, while continuous data were described using means and standard deviations (SD). Variables normally distributed were compared using parametric tests, including analysis of variance, and Student’ T-tests, and with non-parametric tests for comparisons of proportions. Comparison of PLS-DA scores among the children with presumptive TB groups and clinical criteria score groups were performed using the Kruskal–Wallis test with Dunn’s post hoc comparisons. Differences were considered statistically significant when a p-value was < 0.05. Analyses were performed using the SPSS 26 software package (SPSS, Chicago, USA).

### Ethical statement

The study protocol was approved by the ethical review board of the Ethics Committee of the University of Barcelona and the Haiti National Ethics Committee (reference number IRB00003099). Written informed consent was obtained from the children’s parents or legal guardians before enrolment. Sample collection and all experiments were performed in accordance with relevant guidelines and regulations.

## Supplementary Information

Supplementary Information.
