# Supplementary material for: Urine NMR-based TB metabolic fingerprinting for the diagnosis of TB in children
Source: Sci Rep. 2021 Jun 7;11:12006. doi: 10.1038/s41598-021-91545-0 (PMC8184981; doi:10.1038/s41598-021-91545-0)
Supplement: Supplementary file 1 — Supplementary Information. [file 41598_2021_91545_MOESM1_ESM.docx]

Urine NMR-based TB metabolic fingerprinting for the diagnosis of TB in children

Patricia Comella-del-Barrio ^1,2, +^, José Luis Izquierdo-Garcia ^2,3,4, +^, Jacqueline Gautier ^5^, Mariette Jean Coute Doresca^5^, Ramón Campos-Olivas ^6^, Clara M. Santiveri ^6^, Beatriz Muriel-Moreno ^1^, Cristina Prat-Aymerich ^1,2,7^, Rosa Abellana ^8^, Tomas M. Pérez-Porcuna ^9^, Luis E. Cuevas ^10^, Jesús Ruiz-Cabello ^2,3,4,11, ¶^, and José Domínguez ^1,2, ¶,^ *

**Supplementary Figures**

- Supplementary Figure 1
- Supplementary Figure 2
- Supplementary Figure 3

**Supplementary Tables**

- Supplementary Table 1

**Supplementary Figures**

**
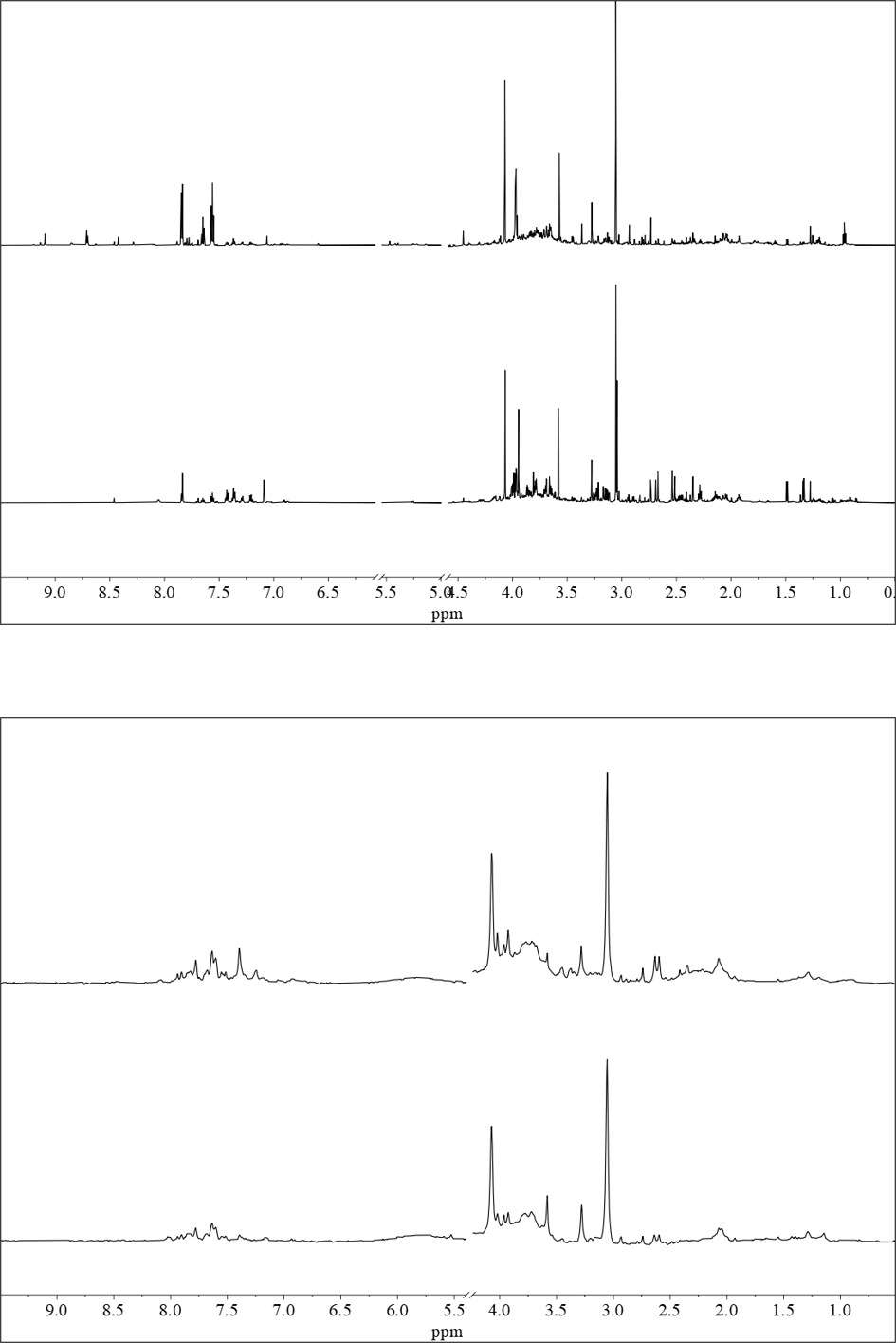
**

**Supplementary Figure 1**. Representative proton (1H) nuclear magnetic resonance (NMR) spectra of urine samples from tuberculosis patients (top) and healthy controls (bottom). (**a**) Urine spectra were acquired using a high field ^1^H NMR Bruker Avance 700 MHz spectrometer. (**b**) Urine spectra were acquired using a low field ^1^H NMR a Magritek Spinsolve 60 Ultra benchtop NMR spectrometer. R rel.3.3.1 (www.R-project.org).

**
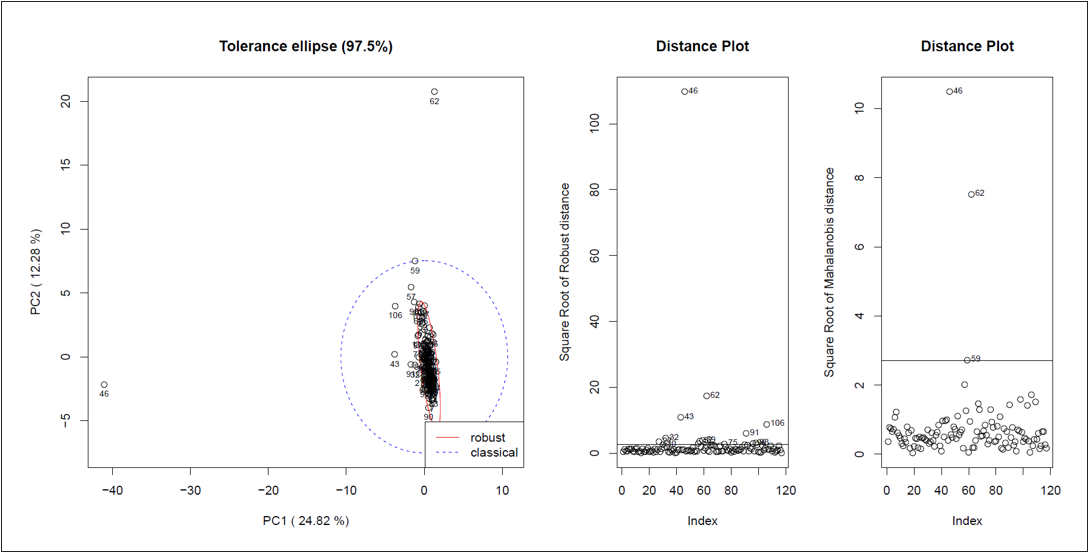
**

**Supplementary Figure 2**. Principal component analysis (PCA) score plot of urine spectra analysed by high field proton (1H) nuclear magnetic resonance (NMR) between tuberculosis and healthy control groups. Two samples (46 and 62) were identified as statistical outliers based on Mahalanobis distance and removed from following analyses. R rel.3.3.1 (www.R-project.org).

**
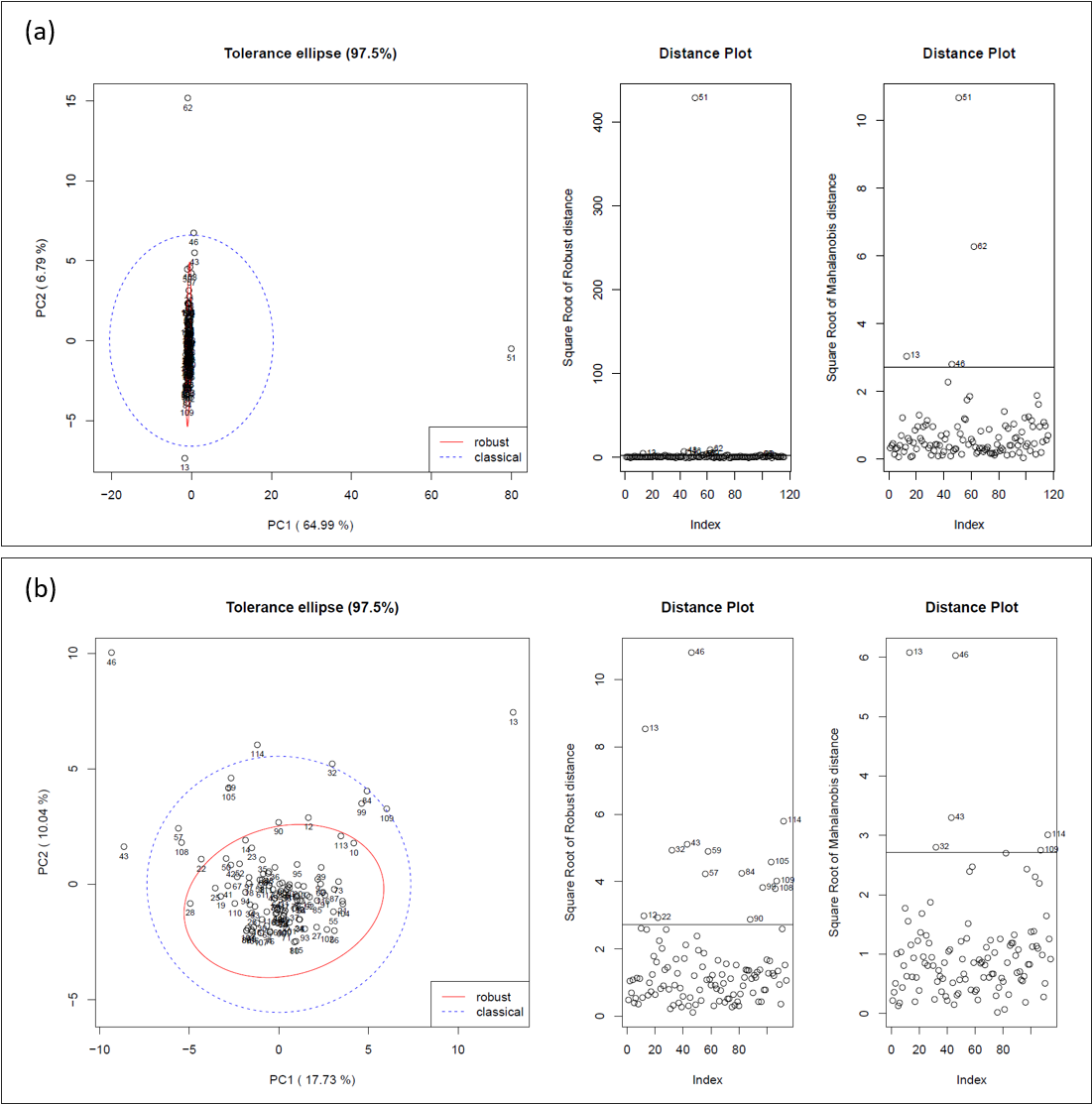
**

**Supplementary Figure 3.** Principal Component Analysis (PCA) score plot of urine spectra analysed by low field proton (1H) nuclear magnetic resonance (NMR) between tuberculosis and healthy control groups. (**a**) Two samples (51 and 62) were identified as statistical outliers based on Mahalanobis distance and removed. (**b**) Second PCA identified another six samples as statistical outliers based on Mahalanobis distance. A total of eight samples (13, 46, 43, 32, 114 and 109) were identified as statistical outliers and removed from following analyses. R rel.3.3.1 (www.R-project.org).

**Supplementary Tables**

**Supplementary Table 1**. Top 15 regions according to the Variable Importance in Projection (VIP) scores for partial least-squares discriminant analysis (PLS-DA) component 1 performed using HF NMR spectra (left) and LF NMR spectra (right). Bold values, statistical significance was determined using a Student’s t-test assuming significant unequal corrected variance with p < 0.05.

| **High field NMR** | | | **Low-field NMR** | | |
| --- | --- | --- | --- | --- | --- |
| **ppm** | **VIP score Comp. 1** | **p -value** | **ppm** | **VIP score Comp. 1** | **p-value** |
| 8,73 | 3,25 | **5,83E-05** | 8,68 | 3,25 | **6,39E-04** |
| 8,61 | 2,62 | 9,34E-02 | 8,11 | 3,21 | 2,00E-01 |
| 8,97 | 2,47 | **2,60E-02** | 2,68 | 3,04 | 2,00E-01 |
| 7,17 | 2,44 | 3,57E-01 | 7,98 | 2,72 | 7,78E-02 |
| 7,77 | 2,32 | **3,06E-03** | 8,29 | 2,69 | 5,69E-01 |
| 8,21 | 2,32 | **5,72E-03** | 3,26 | 2,68 | **1,12E-02** |
| 0,71 | 2,22 | **8,66E-03** | 1,38 | 2,58 | 7,81E-02 |
| 8,09 | 2,15 | 6,11E-01 | 8,69 | 2,52 | **7,55E-03** |
| 9,09 | 2,14 | **4,07E-03** | 8,74 | 2,51 | 5,07E-01 |
| 9,25 | 2,14 | **4,43E-02** | 3,69 | 2,50 | 2,64E-01 |
| 7,37 | 2,05 | 1,38E-01 | 10,00 | 2,49 | 1,41E-01 |
| 4,12 | 2,00 | **1,62E-02** | 9,20 | 2,48 | 9,73E-02 |
| 9,93 | 1,98 | **4,27E-02** | 8,12 | 2,47 | 1,09E-01 |
| 3,12 | 1,93 | 3,45E-01 | 8,77 | 2,37 | 6,18E-01 |
| 2,84 | 1,85 | 8,75E-02 | 9,10 | 2,35 | 6,47E-01 |

NMR, nuclear magnetic resonance; ppm, parts per million, VIP, Variable Importance in Projection.
